# Tumor Necrosis Factor Receptor Associated Factor 6 Is Not Required for Atherogenesis in Mice and Does Not Associate with Atherosclerosis in Humans

**DOI:** 10.1371/journal.pone.0011589

**Published:** 2010-07-14

**Authors:** Peter Stachon, Anna Missiou, Carina Walter, Nerea Varo, Christian Colberg, Dennis Wolf, Maike Buchner, Constantin von zur Mühlen, Katja Zirlik, Christoph Bode, Andreas Zirlik

**Affiliations:** 1 Department of Cardiology, University of Freiburg, Freiburg, Germany; 2 Spemann Graduate School of Biology and Medicine (SGBM), University of Freiburg, Freiburg, Germany; 3 Faculty of Biology, University of Freiburg, Freiburg, Germany; 4 Department of Clinical Chemistry, University of Navarra, Pamplona, Spain; 5 Department of Hematology and Oncology, University of Freiburg, Freiburg, Germany; Karolinska Institutet, Sweden

## Abstract

**Background:**

Tumor necrosis factor receptor-associated factors (TRAFs) are important signaling molecules for a variety of pro-atherogenic cytokines including CD40L, TNF α, and IL1β. Several lines of evidence identified TRAF6 as a pro-inflammatory signaling molecule *in vitro* and we previously demonstrated overexpression of TRAF6 in human and Murine atherosclerotic plaques. This study investigated the role of TRAF6-deficiency in mice developing atherosclerosis, a chronic inflammatory disease.

**Methodology/Principal Findings:**

Lethally irradiated low density lipoprotein receptor-deficient mice (TRAF6^+/+^/LDLR^−/−^) were reconstituted with TRAF6-deficient fetal liver cells (FLC) and consumed high cholesterol diet for 18 weeks to assess the relevance of TRAF6 in hematopoietic cells for atherogenesis. Additionally, TRAF6^+/−^/LDLR^−/−^ mice received TRAF6-deficient FLC to gain insight into the role of TRAF6 deficiency in resident cells. Surprisingly, atherosclerotic lesion size did not differ between the three groups in both aortic roots and abdominal aortas. Similarly, no significant differences in plaque composition could be observed as assessed by immunohistochemistry for macrophages, lipids, smooth muscle cells, T-cells, and collagen. In accord, in a small clinical study TRAF6/GAPDH total blood RNA ratios did not differ between groups of patients with stable coronary heart disease (0.034±0.0021, N = 178), acute coronary heart disease (0.029±0.0027, N = 70), and those without coronary heart disease (0.032±0.0016, N = 77) as assessed by angiography.

**Conclusion:**

Our study demonstrates that TRAF6 is not required for atherogenesis in mice and does not associate with clinical disease in humans. These data suggest that pro- and anti-inflammatory features of TRAF6 signaling outweigh each other in the context of atherosclerosis.

## Introduction

Atherosclerosis, one of the leading causes of morbidity and mortality in Western countries [1], is a chronic inflammatory disease driven by an armada of inflammatory cells and their effector cytokines. A solid body of evidence supports the concept that inflammation promotes atherogenesis at every step from initiation to progression, destabilization, and complication. Although the inflammatory nature of this disease had been uncovered more than a decade ago a genuine anti-inflammatory or immune-modulatory treatment option is still absent in current therapeutic regimens [2]. Along with extensive basic experimental data increasing clinical evidence attribute great potential to such therapeutic strategies. The recent JUPITER trial is an example *par excellence* in that respect [3]. While unselective inhibition of pro-inflammatory cytokines such as CD40L initially held great promise these strategies either proved to have inherent deleterious side effects or appear unfit for long-term treatment likely required by the chronic inflammatory nature of atherosclerotic disease [4]. Selective inhibition of key signaling branch points, however, may overcome some of these limitations and demonstrated feasibility in the treatment of other disorders such as malignancies.

Tumor necrosis factor (TNF) receptor-associated factors (TRAFs) are intracellular adaptor proteins, which channel signaling for members of the TNF-/interleukin-1 (IL-1)-/toll-like-receptor (TLR)-superfamily such as TNFα, CD40L, and IL-1β, proteins known to promote inflammation and atherosclerosis [5], [6], [7]. To date seven TRAFs have been characterized sharing a common C-terminal domain important for binding to upstream receptors and with the exception of TRAF1 sharing a RING-zinc finger for downstream signal propagation [8], [9]. TRAF6, a 63 kDa molecule, was first identified as an adaptor protein of CD40 and independently as a signal transducer for IL-1 [10], [11]. TRAF6 differs from the other TRAFs in recognizing a distinguished amino acid sequence allowing for participation in inflammatory signaling of both the TNFR and IL1/Toll-like receptor pathways [12], [13], [14]. While overexpression of TRAF6 induces NFκB activation, dominant negative mutants of TRAF6 inhibit the NFκB pathway activated by IL-1β but not TNFα[10], [15], [16]. Interestingly, cells with mutant CD40 molecules eliminating the interaction with TRAF6 showed impaired NFκB, JNK, and p38 activation [17]. Similarly, IL-1β-mediated activation of these molecules was completely abrogated in TRAF6-deficient mixed embryonic fibroblasts [18]. Furthermore, expression of exogenous TRAF6 in TRAF6-deficient cells restored NFκB, JNK, and p38 phosphorylation [19]. These data suggest that TRAF6 figures as pro-inflammatory molecule.

Data on the function of TRAF6 in vascular disease are scarce. Human monocytes transfected with TRAF6 binding protein inhibiting the association of TRAF6 with CD40, failed to activate ERK1/2, IKK, and cytokine production after stimulation with CD40L [20]. Donners *et al.* demonstrated that mice carrying a mutated TRAF6 with selective incapability of CD40 binding develop decreased neointima formation in a carotid injury model. Similar findings were also obtained in rabbits upon transfection of a plasmid containing the dominant negative form of TRAF6 [21], [22]. Our group recently demonstrated overexpression of several TRAFs including TRAF6 in Murine and human atherosclerotic lesions [23]. Based on these data we hypothesized that TRAF6 promotes atherogenesis in mice and associates with atherosclerosis and its complications in humans.

## Results

### Fetal liver cell transplantation successfully reconstitutes hematopoeietic cells

**Figure 1:**
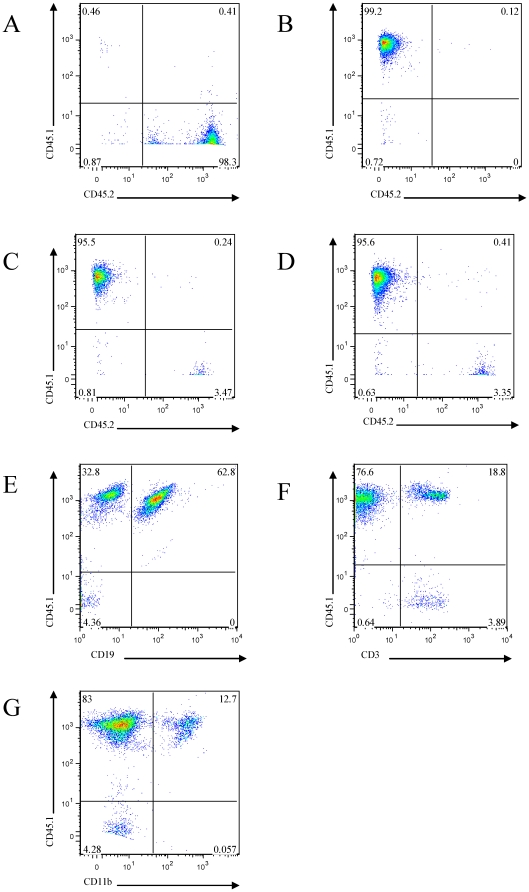
Successful reconstitution of peripheral blood cells by fetal liver cell transplantation. LDLR^−/−^ mice (CD45.2-positive/CD45.1-negative, A) were lethally irradiated (2×450 cGy) and reconstituted with fetal liver cells of 6–8 week old CD45.1-positive/CD45.2-negative mice (B). After an interval of 4 weeks, peripheral blood cells were immunostained with CD45.1-PE and CD45.2-FITC (exemplary donors are shown in C and D) or CD45.1-PE in combination with CD19-PECy (B-cell marker, E), CD3-APC (T-cell marker, F), and CD11b-FITC (monocytic marker, G) antibodies and analyzed by FACS.

**Figure 2:**
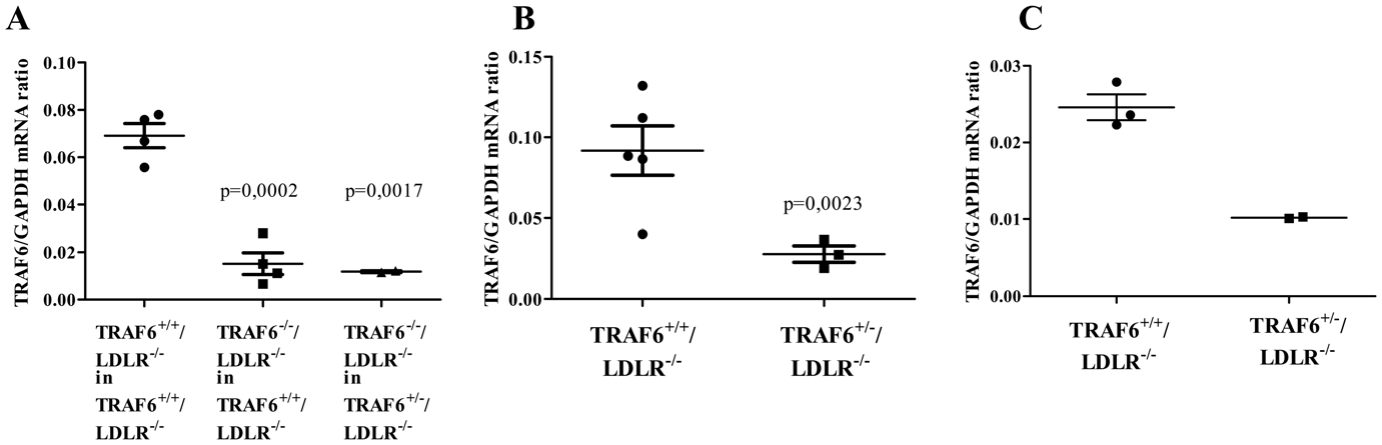
Mice reconstituted with TRAF6-deficient fetal liver cells and TRAF6 heterozygous mice express less TRAF6 than controls. Lethally irradiated 6 week old TRAF6^+/+^/LDLR^−/−^ mice received TRAF6-deficient (N = 4) or competent fetal liver cells (N = 4), TRAF6^+/−^/LDLR^−/−^ mice received TRAF6-deficient fetal liver cells (N = 2) only. RNA was isolated from spleens. Ratios of TRAF6/GAPDH mRNA as assessed by quantitative RT-PCR are shown as mean±SEM (A). RNA was isolated from spleens (B) and aortas (C) of TRAF6^+/+^/LDLR^−/−^ (N = 5 and 3) TRAF6^+/−^/LDLR^−/−^ mice (N = 3 and 2). Ratios of TRAF6/GAPDH mRNA as assessed by quantitative RT-PCR are shown as mean±SEM.

Exploration of atherogenesis in TRAF6-deficient mice was hampered by the limited viability of homozygous TRAF6-deficient mice [15], [16]. To circumvent this limitation we performed fetal liver cell (FLC) transplantations. To test the hypothesis that TRAF6 in FLC-derived cells contributes to atherogenesis, FLC from TRAF6^−/−^/LDLR^−/−^ and TRAF6^+/+^/LDLR^−/−^ were transplanted into TRAF6^+/+^/LDLR^−/−^ mice. To explore a putative additional effect of TRAF6 in resident cells such as endothelial cells and smooth muscle cells, a third group of chimera was generated by transplanting FLC from TRAF6^−/−^/LDLR^−/−^ into TRAF6^+/−^/LDLR^−/−^ mice. In this model, transplantation of FLC from CD45.2 mice into CD45.1 recipients validated successful reconstitution of CD3-, CD19-, and CD11b-positive cells in our hands (Fig. 1). Furthermore, spleens of both TRAF6^+/+^/LDLR^−/−^ and TRAF6^+/−^/LDLR^−/−^ mice receiving TRAF6-deficient FLC showed impaired TRAF6/GAPDH mRNA expression as assessed by quantitative PCR, also indicating successful reconstitution (Fig. 2A).

To verify the assumption that TRAF6 heterozygous mice express lower levels of TRAF6, we also analyzed TRAF6 expression in spleens and aortas of TRAF6^+/+^/LDLR^−/−^ and TRAF6^+/−^/LDLR^−/−^ animals without transplantation. In both cases, TRAF6 heterozygous mice expressed lower TRAF6/GAPDH mRNA ratios (Fig. 2B and C).

### TRAF6 deficiency attenuates weight gain and plasma cholesterol increase on high cholesterol diet

**Figure 3:**
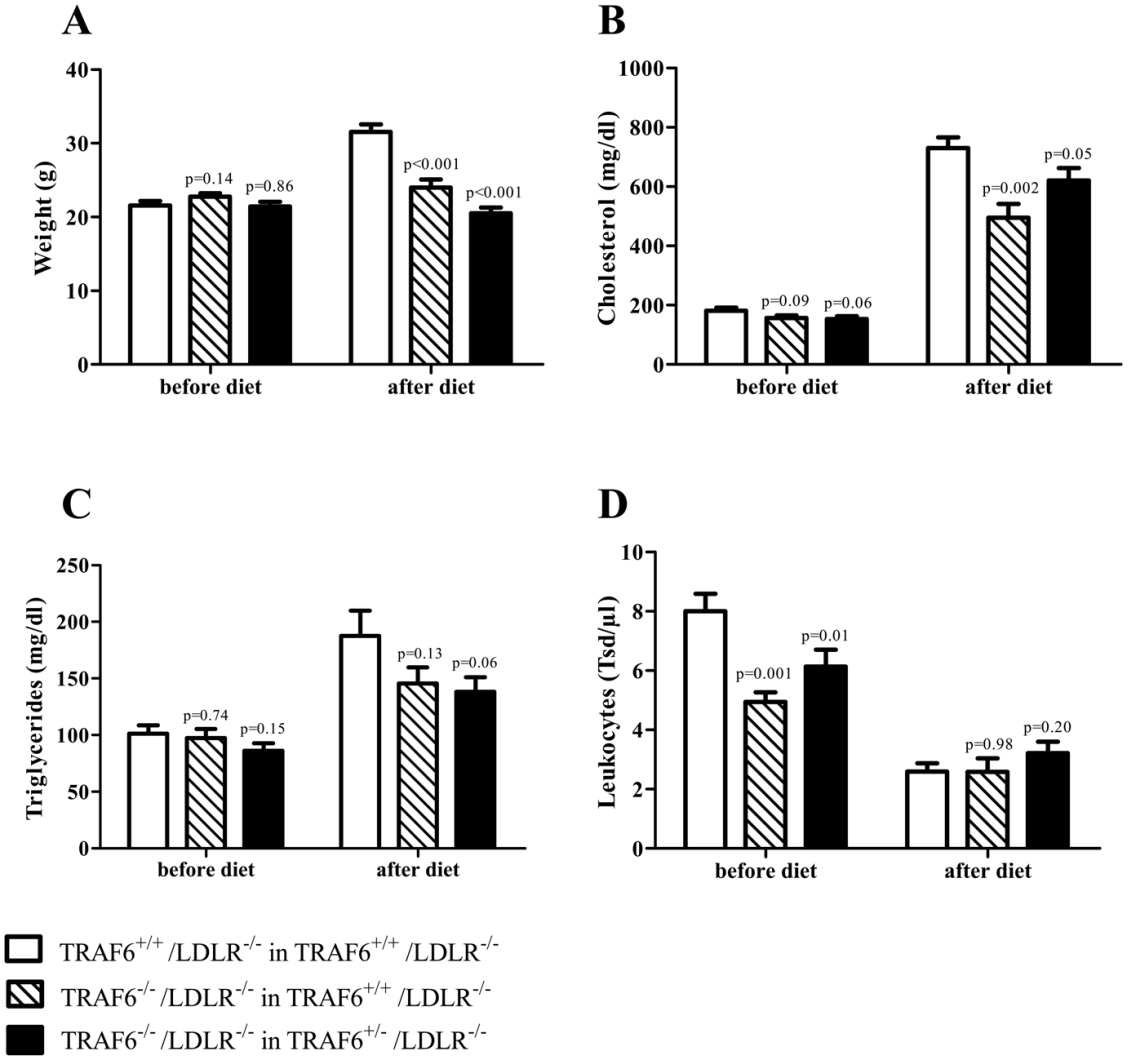
Weights, cholesterol-, and leukocyte levels before and after high cholesterol diet. Lethally irradiated 6 week old TRAF6^+/+^/LDLR^−/−^ mice received TRAF6-deficient (hatched bars, N = 22) or competent fetal liver cells (white bars, N = 27), TRAF6^+/−^/LDLR^−/−^ mice received TRAF6-deficient fetal liver cells (black bar, N = 23) only. Subsequently, all groups consumed high cholesterol diet (HCD) for 18 weeks. Weights (A), plasma cholesterol levels (B), triglycerides (C), and leukocyte counts (D) were taken before and after HCD.

After 4 weeks allowed for reconstitution, mice consumed a high cholesterol diet for 18 weeks. At the beginning of the study no significant difference in body weight, plasma cholesterol, triglyceride levels, and phenotype was observed (Fig. 3A–C). However, all groups receiving TRAF6-deficient FLC had significantly lower peripheral leukocyte counts, an effect that could no longer be detected after the feeding period (Fig. 3D). No difference could be detected as to the percentage of the T cell subtypes CD4, CD8, and T_regs_, of the inflammatory monocyte subset Ly6C^high^ monocytes, and the percentage of B cells (Table 1). At the end of the study TRAF6^+/+^/LDLR^−/−^ mice reconstituted with TRAF6-deficient cells (24.0±1.1 g, N = 22) weighed significantly less than TRAF6^+/+^/LDLR^−/−^ mice receiving TRAF6-competent FLC (31.5 g±1.1 g, N = 27. p<0.0001). TRAF6^+/−^/LDLR^−/−^ mice reconstituted with TRAF6-deficient cells did not gain weight at all on HCD (20.5 g±0.7 g, N = 24, p<0.0001, Fig. 3A).

**Table 1:** Subtypes of blood leukocytes.

	TRAF6^+/+^/LDLR^−/−^	TRAF6^+/−^/LDLR^−/−^	TRAF6^+/+^/LDLR^−/−^ in TRAF6^+/+^/LDLR^−/−^	TRAF6^−/−^/LDLR^−/−^ in TRAF6^+/+^/LDLR^−/−^	TRAF6^−/−^/LDLR^−/−^ in TRAF6^+/−^/LDLR^−/−^
CD4+% of T- cells	51.2±6.7	55.9±3.5	65.9±1.1	63.6±2.5	65.0±1.3
CD8+% of T- cells	37.0±2.6	38.1±3.5	28.2±1.0	28.3±5.0	28.5±3.4
T- reg% of T- cells	3.7±2.3	3.4±2.3	3.4±2.4	4.5±2.0	6.6
B- cells% of leukocytes	23.9±13.0	23.8±4.7	20.6±2.2	19.5±4.1	20.9
Inflammatory monocytes % of total monocytes	65.6±19.7	67.4±13.8	65.0±12.2	63.4±23.4	70.6

Also, TRAF6^+/+^/LDLR^−/−^ mice receiving TRAF6-deficient FLC (496±46 mg/dl, N = 21) had significantly lower plasma cholesterol levels than those receiving TRAF6-competent FLC (730 mg/dl±36 mg/dl, N = 23, p = 0.002) after 18 weeks of HCD while no significant difference in cholesterol levels could be detected when compared with TRAF6^+/−^/LDLR^−/−^ mice receiving TRAF6-deficient FLC (620 mg/dl±41 mg/dl, N = 22, p = 0,0513 Fig. 3B). There were no significant differences in triglycerides between the tested groups.

### TRAF6 deficiency does not alter atherosclerotic lesion size and plaque composition in aortic roots

**Figure 4:**
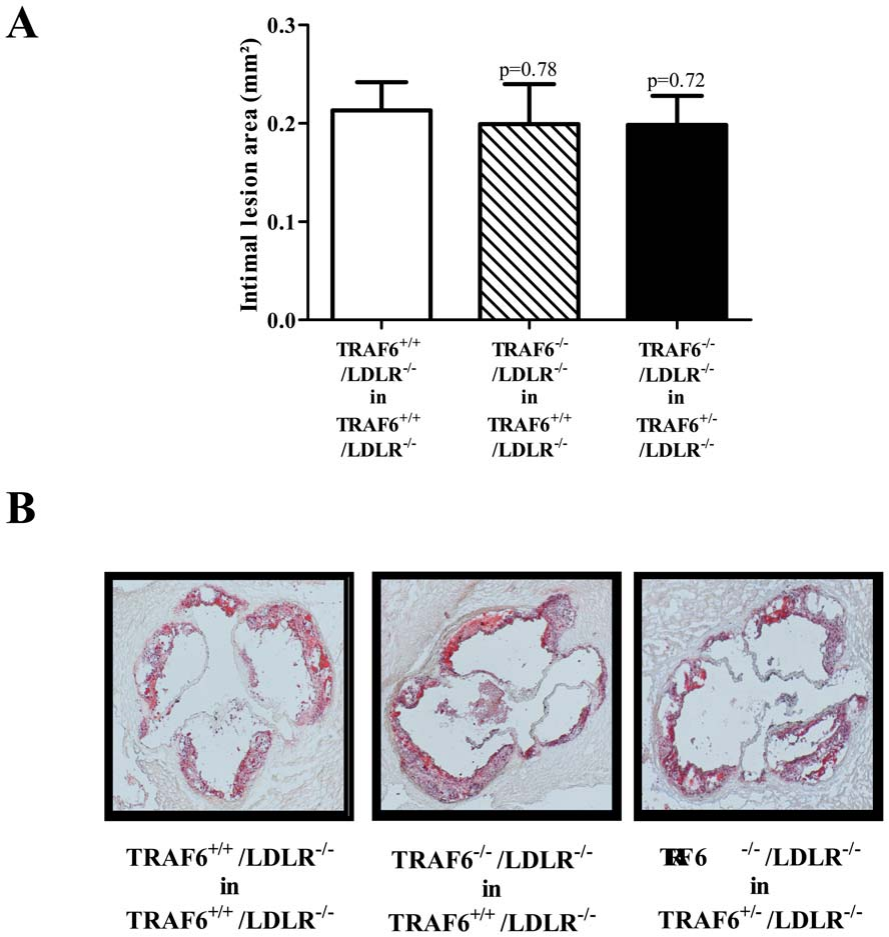
TRAF6 deficiency does not modulate atherogenesis in mice. Lethally irradiated 6 week old TRAF6^+/+^/LDLR^−/−^ mice received TRAF6-deficient (hatched bars, N = 21) or competent fetal liver cells (white bars, N = 21), TRAF6^+/−^/LDLR^−/−^ mice received TRAF6-deficient fetal liver cells (black bars, N = 22) only. Subsequently, all groups consumed high cholesterol diet (HCD) for 18 weeks. Intimal lesion area of the atherosclerotic plaques in aortic roots was quantified. Pooled mean intimal lesion area ± SEM are shown as graphs in the upper panel (A), representative sections stained with oil red O below (B).

**Figure 5:**
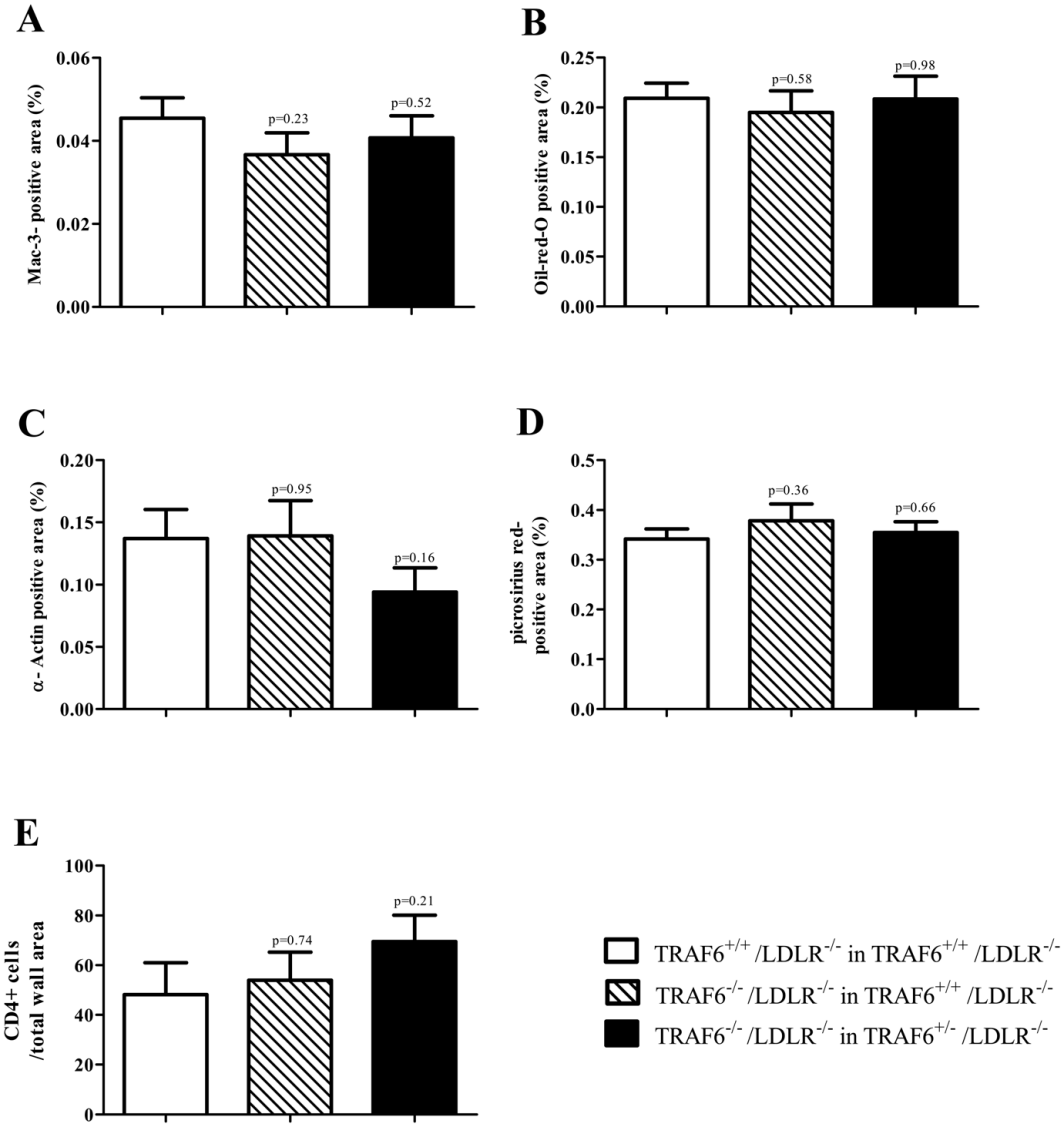
TRAF6 deficiency does not alter plaque composition. Lethally irradiated 6 week old TRAF6^+/+^/LDLR^−/−^ mice received TRAF6-deficient (hatched bars, N = 21) or competent fetal liver cells (white bars, N = 21), TRAF6^+/−^/LDLR^−/−^ mice received TRAF6-deficient fetal liver cells (black bars, N = 22) only. Subsequently, all groups consumed high cholesterol diet (HCD) for 18 weeks. Sections of the aortic roots were analyzed for macrophage- (A), lipid- (B), smooth muscle cell- (C), collagen (D), and T cell-content (E). Mac-3-, oil-red-O-, α-actin-, picrosirius red, and CD4-positive staining in per cent of total wall area is displayed as mean±SEM.

Intimal lesion size in the aortic roots of TRAF6^+/+^/LDLR^−/−^ (0.199 mm^2^±0.04 mm^2^, N = 21, p = 0.2825) and TRAF6^+/−^/LDLR^−/−^ mice (0.198 mm^2^±0.03 mm^2^ N = 22, p = 0.7178) reconstituted with TRAF6-deficient FLC did not differ compared with TRAF6^+/+^/LDLR^−/−^ mice receiving TRAF6-competent FLC (0.213 mm^2^±0.03 mm^2^, N = 21, Fig. 4). Next we tested whether TRAF6, though not altering lesion size, modulates cellular composition of the atherosclerotic plaque. TRAF6 deficiency did not change lesional macrophage-, lipid-, collagen-, smooth muscle cell-, or T-cell- content, suggesting no relevant effect of TRAF6 on atherosclerotic plaque formation (Fig. 5).

### TRAF6 deficiency does not modulate atherosclerosis in the abdominal aorta

**Figure 6:**
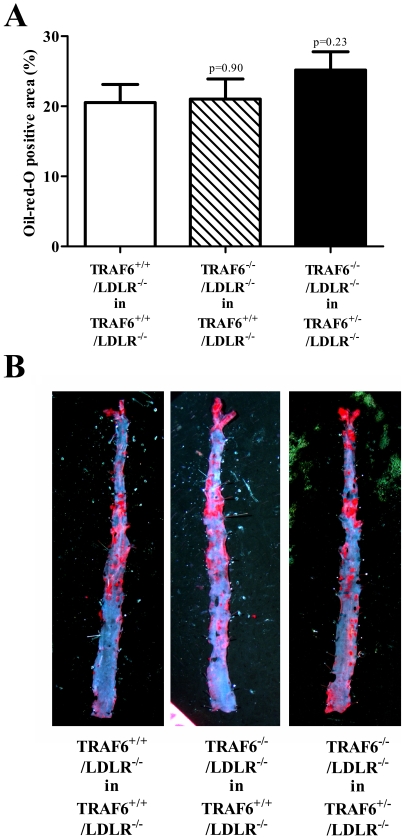
TRAF6 deficiency does not alter lipid deposition in the abdominal aorta. Lethally irradiated 6 week old TRAF6^+/+^/LDLR^−/−^ mice received TRAF6-deficient (hatched bars, N = 10) or competent fetal liver cells (white bars, N = 10), TRAF6^+/−^/LDLR^−/−^ mice received TRAF6-deficient fetal liver cells (black bars, N = 10) only. Subsequently, all groups consumed high cholesterol diet (HCD) for 18 weeks. Abdominal aortas were fixed in formalin, pinned, and stained with oil red O to detect lipid deposition. Oil red O-positive staining in per cent of total area is shown as mean±SEM in the upper panel (A), representative images are shown below (B).

Since effects on atherogenesis may be site- and stage-specific we also analyzed lesion formation *en face* in the abdominal aortas [24]. As expected, lesion formation was reduced in abdominal aortas, a phenomenon known in animals that underwent irradiation [25]. Again, TRAF6 deficiency did not alter lesion size as assessed by Oil red O staining (Fig. 6).

### TRAF6 deficiency does modulate the inflammatory gene expression of macrophages

**Figure 7:**
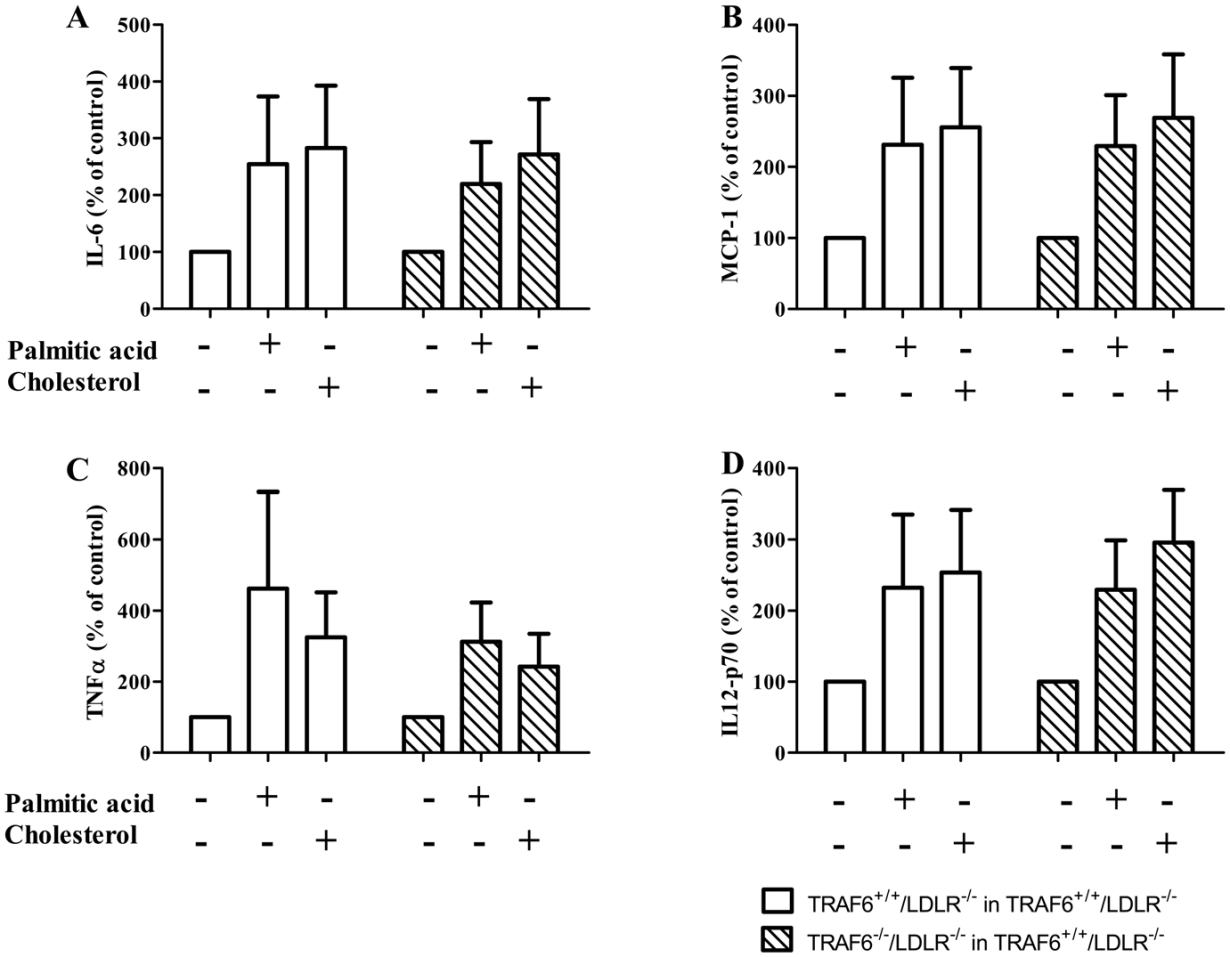
TRAF6 deficiency does not modulate inflammatory reactivity of macrophages toward cholesterol and palmitic acid. Bone marrow-derived macrophages were isolated from 6 week old TRAF6^+/+^/LDLR^−/−^ mice receiving TRAF6-deficient (hatched bars, N = 4) or competent fetal liver cells (white bars, N = 4), were stimulated with 4 mg/ml cholesterol or 0.75 µM palmitic acid, and assayed for expression of IL-6 (A), MCP-1 (B), TNFα (C), and IL12-p70 (D) by cytometric bead array.

Since TRAF6^+/+^/LDLR^−/−^ and TRAF6^+−^/LDLR^−/−^ mice showed decreased levels of cholesterol at the end of the study (Fig. 3B) a putative difference in macrophage reactivity toward cholesterol or fatty acids could explain why we observed similar atherosclerotic lesion formation in these mice compared with respective wild-type controls and could therefore mask a phenotype. Thus, we isolated bone marrow-derived macrophages from TRAF6^+/+^/LDLR^−/−^ mice reconstituted with either TRAF6-deficient or -competent FLC and stimulated these with cholesterol and palmitic acid. Interestingly, macrophages from both groups expressed similar amounts of interleukin-6 (IL-6), monocyte chemoattractant protein-1 (MCP-1), tumor necrosis factor alpha (TNFα), and interleukin-12 (IL-12) as assessed by cytometric bead array (Fig. 7), suggesting that our observations are not due to a TRAF6-dependent difference in inflammatory reactivity of macrophages.

### TRAF6 mRNA expression in blood does not associate with acute or chronic coronary heart disease in humans

**Figure 8:**
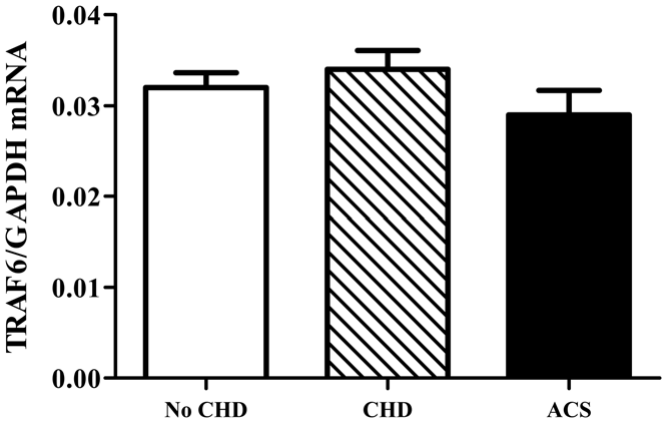
TRAF6 expression in blood does not associate with acute or chronic coronary heart disease. 325 patients undergoing coronary angiography were divided into three groups: no coronary heart disease (No CHD, N = 77), stable coronary heart disease (CHD, N = 178), and acute coronary syndromes (ACS, N = 70). TRAF6 and GAPDH mRNA was analyzed by quantitative real-time PCR in total blood RNA. Results are presented as mean±SD computed from the average measurements obtained from each group.

Since we previously demonstrated overexpression of TRAF6 protein in human plaques, we tested the hypothesis that TRAF6 expression associates with acute or chronic coronary heart disease in humans [23]. Therefore, we measured TRAF6 mRNA in blood of a total of 325 patients undergoing coronary angiography categorized into three groups: no coronary heart disease (No CHD, N = 77), stable coronary heart disease (CHD, N = 178), and acute coronary syndrome (ACS, N = 70). The baseline characteristics of the study groups have been previously published [26] and are shown in table 2. Gender and BMI did not significantly differ among the groups, while patients were older in the CHD group and had more traditional cardiovascular risk factors in the CHD and ACS groups. TRAF6/GAPDH mRNA ratios did not differ between the groups corroborating the data gathered in mice (Fig. 8).

**Table 2:** Demographic and clinical characteristics of study participants.

	No CHD (n = 77)	CHD (n = 178)	ACS (n = 70)
Age (years)	62±1	65±0,6*	64±1
BMI (kg/m2)	28.2±0.5	27.5±0.3	27.8±0.5
% men	71	83	79
% diabetes	7,6	24.6 *	25.7 *
% hypertension	12,8	40.2 *	15.5 ∫
% smokers	9,1	27.4 *	14
SBP	131±1	131±1	133±2
DBP	77±1	77±1	79±1
Glucose	110±4	114±3	120±6
Cholesterol	202±7	182±21 *	196±9
Triglycerides	150±19	151±9	171±18
LDL	116±5	96±4 *	99±7 *
VLDL	35±4	33±2	40±3 ∫
HDL	52±3	48±1	53±5
Creatine kinase	109±9	117±12	709±155 *,∫
Pro-BNP	389±145	534±98	1330±509 *,∫

*, p<0.05 vs controls.

∫, p<0.05 vs CHD.

## Discussion

The current study presents the novel and unexpected finding that TRAF6 deficiency on FLC-derived cells does not alter atherogenesis in either TRAF6^+/+^/LDLR^−/−^ or TRAF6^+/−^/LDLR^−/−^ mice. Our data challenge the common view of TRAF6 as pro-inflammatory signaling molecule in the context of atherosclerosis.

Multiple reports identified TRAF6 as positive regulator of CD40L- and IL-1 but not TNFα-induced NFκB signaling [10], [15], [16], [17], [27] *in vitro*. Furthermore, TRAF6 is essential for signaling via the toll-like receptors 2, 5, 7, and 9 but not -3 [27]. *In vivo* evaluation of the genetic deficiency in TRAF6 confirmed its role in CD40-, IL-1-, and LPS signaling and phenotypically resulted in severe osteopetrosis [15], [16]. In line with a pro-inflammatory function of TRAF6 Kobayashi *et al.* reported impaired maturation of TRAF6-deficient dendritic cells *in vitro* and *in vivo* [18], a cell type crucially involved in adaptive immunity by presenting antigens to T cells [28]. Similarly, two previous reports implicated TRAF6 with neointima formation [21], [22]. In contrast, our data suggest no role for TRAF6 in the chronic inflammatory disease atherosclerosis. Although TRAF6 deficiency impaired weight gain and decreased plasma cholesterol levels, attributes that would commonly favor smaller atherosclerotic lesions, TRAF6 deficiency did not reduce plaque formation. These data are in accord with our previous report demonstrating no modulation of atherogenesis for the TRAF6 cognate receptor CD40 [29]. Interestingly, Akiyama *et al.* reported a disrupted thymic structure, reduced numbers of regulatory T cells, and an autoimmune phenotype with inflammatory infiltrates in most organs in TRAF6-deficient mice, suggesting rather an anti-inflammatory role for TRAF6 [30]. These opposing results demonstrate that TRAF functions may be diverse and depend indeed on stimulus and cell type warranting a disease-based evaluation [8]. Of note, TRAF6-competent and -deficient macrophages responded similarly to stimulation with cholesterol and acid palmitic acid suggesting that our findings are not due to a TRAF6-dependent difference in inflammatory reactivity.

Recently, Lutgens *et al.* found reduced atherosclerotic lesion formation in mice lacking the binding site for TRAF6 on CD40 in monocytes/macrophages [31]. Our data are not in contrast with this finding. Specific CD40-TRAF6 signaling may very well be pro-atherogenic while overall signaling through TRAF6 by various upstream receptors may have no net effect on atherogenesis. Indeed, several of the upstream binding partners such as CD40L, IL1β, and TLR4 are well known propagators of atherogenesis [6], [7], [32]. However, TRAF6 also interacts with proteins known to attenuate atherosclerotic lesion formation: recently it could be shown, that TLR5- deficient mice develop a metabolic syndrome [33]. Moreover, *Miller et al*. reported an anti-atherogenic effect of IL-33 [34], which also signals through TRAF6 [35]. Thus, TRAF6 may initiate anti-inflammatory signals outweighing its pro-inflammatory attributes. In line with this notion, we previously demonstrated increased expression of IL-6, MCP-1, and IL-8 in the supernatants of TRAF6-silenced human umbilical vein endothelial cells (HUVEC) upon stimulation with CD40L and TNFα in endothelial cells and bone marrow-derived macrophages [23].

Several studies implicate TRAF6 in the recruitment and function of mononuclear cells [21], [31]. Mukundan *et al.* reported that TRAF6 is crucial in CD40-mediated activation of ERK1/2 NFκB, and inflammatory cytokine production [20]. However, these findings were not reflected by a change in lesional macrophage content in our study. Not only did plaque size not differ between the study groups but also lesion composition was similar in the present study, again suggesting that the pro-inflammatory features of TRAF6 are counterbalanced in atherogenesis.

Since we previously observed increased expression of TRAF6 in human carotid plaques we tested whether TRAF6 mRNA levels in blood associate with chronic or acute coronary heart disease. We observed no significant difference in TRAF6 expression between the tested groups: no coronary heart disease (no CHD), stable coronary heart disease (CHD) and acute coronary syndrome (ACS), corroborating our findings obtained in mice.

Our study has several limitations: First, we cannot rule out that the lower cholesterol levels observed in animals receiving TRAF6-deficient bone marrow mask a putative effect of TRAF6 deficiency. This is, however, unlikely since previous reports mainly suggest a pro-inflammatory function of TRAF6 and therefore one would expect reduced levels of atherosclerosis in mice deficient in TRAF6. Lower cholesterol levels also predispose for smaller lesions. Therefore, these should not impair the results of our study. Furthermore, we found no evidence for a TRAF6-dependent difference in inflammatory reactivity of macrophages, which could also mask a putative effect of TRAF6 in our model. Secondly, since γ-irradiation itself profoundly influences the development of atherosclerotic lesions we cannot rule out that this affects our results [25]. However, all groups were treated equally. Therefore, differences should still be detected between the groups.

In summary, we present the novel and surprising finding that TRAF6 deficiency does not influence atherogenesis in mice and does not associate with atherosclerosis in humans. Therefore, overall targeting of TRAF6 may not be a promising treatment strategy for atherosclerosis and probably also other chronic inflammatory diseases.

## Materials and Methods

### Genotyping and housing of Mice

All animal procedures were approved by the Animal Board of Freiburg (Regierungspräsidium Freiburg, permit number G05/41). TRAF6^+/−^ mice were kindly provided by Dr. T. W. Mak and fully backcrossed to C57/BL6 background as verified by background strain characterization at Jackson laboratories. Mice were crossbred with LDLR^−/−^ mice (Jackson) to generate TRAF6^+/−^/LDLR^−/−^ and TRAF6^+/+^/LDLR^−/−^ mice. Genotyping of each mouse used polymerase chain reaction employing the following primers: LDLR, 5′-CCA TAT GCA TCC CCA GTC TT-3′ (common primer), 5′-GCG ATG GAT ACA CTC ACT GC-3′ (wild-type primer), 5′-AAT CCA TCT TGT TCA ATG GCC GAT C-3′ (mutant primer); TRAF6, 5′-CTG CAG TGA AAG ATG ACA GCG TGA GT-3′ (wild-type) ; 5′-CCA AGT GCC CAG CGG GGC TGC TAA AG -3′ (neo), 5′-ACG GAA GCA AGC CTC TGT TCA TAC CG-3′ (common). All mice were housed under specific pathogen-free conditions.

### Fetal Liver Cell Transplantation

Fetal livers were obtained 17 days after conception from fetuses of TRAF6^+/−^/LDLR^−/−^ mice. One arm was used for genotyping. Four week-old male TRAF6^+/+^/LDLR^−/−^ and TRAF6^+/−^/LDLR^−/−^ recipient mice were lethally irradiated with two doses of 450 cGy at a 6 h interval (Gammacell Exactor 40). Fetal livers were suspended with a pipette, filtered through a 100 µm cell strainer (BD bioscience), centrifuged, resuspended, and injected at 10^6^ cells/300 µl into the tail vein. Transplanted mice received chow diet for four weeks allowing for reconstitution. To verify reconstitution, fetal liver cells from CD45.1 mice were transplanted into CD45.2 mice and reconstitution rates were assessed by FACS after 4 weeks.

### Fluorescence-activated cell sorter analysis (FACS)

FACS analysis was performed as described previously [36].

### High cholesterol diet and harvest

After four weeks of reconstitution recipient mice consumed a high-cholesterol diet (HCD) for 18 weeks (Ssniff based on Research Diets D12108). Subsequently, mice were euthanized, hearts and aortas were removed, and histologically prepared as described previously [29], [36].

### Lipoprotein measurement and leukocyte count

Blood samples were collected by retro-orbital puncture before and at the end of HCD after an overnight starvation. Serum total cholesterol and triglyceride concentrations were assayed by commercially available enzymatic assays according to the manufacturer's protocols (CHOL-H L and Triglyceride L-Type from WAKO).

### RNA extraction, cDNA synthesis, and quantitative real-time PCR

Harvested organs were stored in RNAlater (Qiagen) at −80°C. RNA was extracted from murine aortas and spleens using TRIzol Reagent (invitrogen) utilizing a modified protocol. Homogenization was performed using a rotor-stator dispergator (IKA). 1 µg of total RNA was transcribed into cDNA using the Transcriptor 1^st^ Strand cDNA Synthesis Kit (Roche). The cDNA obtained was subjected to quantitative real-time PCR with a Roche LightCycler 480 using the LightCycler 480 SYBR Green I Master (Roche). mGapdh served as endogenous control. Amplification of potential genomic DNA contamination was ruled out by using intron-spanning primer pairs and subsequent reassurance through melting curve analysis. The following primers were employed: mGapdh: 5′-TGC ACC ACC AAC TGC TTA G-3′ (foward) and 5′-GAT GCA GGG ATG ATG TTC-3′ (reverse), mTraf-6: 5′-TGT TCT TAG CTG CTG GGG TGT-3′ (foward) and 5′-GAA GGA GCT GGA GAG GTT CC-3′ (reverse). For normalization, the ratio of mTRAF6/mGAPDH was calculated. P values lower than 0.05 were considered significant.

### Oil red O staining for lipids of cryostat sections and abdominal aortas

Frozen sections were air dried, fixed in 10% formalin for 10 min, washed, submerged in 100% propyleneglycol (Fisher scientific), incubated in oil red O (Sigma-Aldrich) for 25 min at 60°C, dipped into 0.25% ammonia H_2_O (EM Science), and coverslipped with glycerol gelatine (Sigma-Aldrich). Abdominal aortas were fixed with 10% formalin, opened longitudinally, pinned, stained with oil red O solution (2.5 h, RT), and washed with 85% propylene glycol.

### Immunohistochemistry

Cryostat sections (6 µm) of mouse aortic roots were air-dried, fixed in acetone at −20°C, incubated with 0.3% H_2_O_2_, blocked with 4% rabbit serum (Vector Laboratories), incubated with primary antibodies (anti-mac-3, anti-α-actin, and anti-CD4 from Pharmingen), incubated with corresponding secondary antibodies (Vector Laboratories and Sigma-Aldrich), washed, incubated with avidin-biotin complex (Vector Laboratories), developed with 3-amino-9-ethylcarbazole (DAKO), counterstained with hematoxylin (Sigma-Aldrich), and coverslipped with glycerol gelatine (Sigma-Aldrich) as described previously [29]. Controls for specificity used IgG controls (Pharmingen, Dako).

### Picrosirius Red Staining for Type I Collagen

Air dried and formalin-fixed frozen sections were incubated for 3 h in 0.1% solution of picrosirius red (Polysciences) in saturated aqueous picric acid (Sigma-Aldrich). Slides were rinsed twice in 0.01 N HCl and distilled water, dehydrated in 70%, 95%, 100% ethanol, incubated in xylene, and mounted in Permount (Vector Laboratories). Picrosirius red staining was analyzed by polarization microscopy (Edmund Industrial Optics).

### Macrophage preparation and stimulation with free fatty acids and cholesterol

6 weeks after transplantation mice were euthanized and bones were removed. Bone marrow was flushed out, cells were cleaned up using ficoll (Biochrom AG, Biocoll Separating Solution), and differentiated to macrophages with 50 ng/ml and subsequently 25 ng/ml M-CSF for 3 days each. Finally, macrophages were stimulated after 24 h starvation with 0.75 µM palmitic acid (Sigma Aldrich) respectively 4 mg/ml cholesterol diluted in ethanol and BSA (Bovine Serum Albumin). The appropriate amount of ethanol and BSA was added to the control. Supernatant was collected and analyzed with cytometric bead array as previously described [25].

### Data analysis

Morphometric calculations of the tissue sections were analyzed by a blinded observer using image pro plus 5.1 (MediaCybernetics). Data were presented as mean±SEM. Comparison of the respective study groups used the Student's two-tailed t-test. The p-value refers to the control group and P<0.05 was considered statistically significant.

### Clinical study

325 patients undergoing coronary angiography were included in the Tumor Necrosis Factor Receptor associated factors in Cardiovascular Risk Study (TRAFICS) approved by the local Institutional Review Board (ethic committees: Ethikkommission der Albert- Ludwigs- Universität Freiburg, permit numbers EK 57/06 and EK 379/09). After written informed consent, blood was drawn from all patients and total blood RNA was isolated by Qiagen PAXgene blood RNA kit according to the manufacturer's instructions. Demographic and clinical characteristics were documented. Patients were divided into three groups: no coronary heart disease (No CHD), stable coronary heart disease (CHD), and acute coronary syndrome (ACS). 1 µg RNA was transcribed into cDNA with use of the transcriptor 1st strand cDNA synthesis kit (Roche). The cDNA obtained was subjected to quantitative real time-PCR with a Roche Light Cycler using the Light Cycler 480 SYBR Green I Master (Roche). As endogenous control, GAPDH was employed. Conditions for quantification of TRAF6 mRNA were 5′-TTG TGC TAG TGC CCT CGA GAA-3′ (forward) and 5′-CTG GAG GAA AAA CTG GGG TGA-3′ (reverse), 45 cycles of 10 s at 95°C, 6 s at 60°C (57°C), and 7 s (10 s) at 72°C. Conditions for quantification of GAPDH mRNA were: 5′-GAA GGT GAA GGT CGG AGT C-3′ (forward) and 5′-GAA GAT GGT GAT GGG ATT TC-3′ (reverse), 45 cycles of 10 s at 95°C, 6 s at 57°C, 10 s at 72°C. For normalization, the ratio of TRAF6/GAPDH copy numbers was calculated. Only real-time PCRs with an efficiency >1.9 and an error <0.05 were analyzed. Statistical analysis was performed with SPSS for Windows. Normal distribution of variables was tested with the Shapiro Wilks test. Differences across groups were compared by ANOVA followed by the Bonferroni post hoc test for normal variables and the Kruskal Wallis for non-normal variables. Results are presented as mean ± standard deviation.
